# Ultrasonography of lower limb vascular angiopathy and plaque formation in type 2 diabetes patients and finding its relevance to the carotid atherosclerotic formation

**DOI:** 10.12669/pjms.301.3907

**Published:** 2014

**Authors:** Jun Duan, Chenglong Zheng, Kuo Gao, Meina Hao, Lin Yang, Dandan Guo, Jingping Wu, Yan Tian, Xueni Song, Jian Liu, Shuwen Guo, Ghulam Murtaza, Min Zheng

**Affiliations:** 1 Jun Duan, Department of Ultrasound, China-Japan Friendship Hospital, Beijing, 100029, P.R. China.; 2 Chenglong Zheng, Beijing University of Chinese Medicine, 11 Bei San Huan Dong Lu, ChaoYang District, Beijing 100029, P. R. China.; 3 Kuo Gao, Department of Ultrasound, China-Japan Friendship Hospital, Beijing, 100029, P.R. China.; 4 Meina Hao, Beijing University of Chinese Medicine, 11 Bei San Huan Dong Lu, ChaoYang District, Beijing 100029, P. R. China.; 5 Lin Yang, Department of Ultrasound, China-Japan Friendship Hospital, Beijing, 100029, P.R. China.; 6 Dandan Guo, Department of Ultrasound, China-Japan Friendship Hospital, Beijing, 100029, P.R. China.; 7 Jingping Wu, Department of Ultrasound, China-Japan Friendship Hospital, Beijing, 100029, P.R. China.; 8 Yan Tian, Department of Ultrasound, China-Japan Friendship Hospital, Beijing, 100029, P.R. China.; 9 Xueni Song, Department of Ultrasound, China-Japan Friendship Hospital, Beijing, 100029, P.R. China.; 10 Jian Liu, Department of Ultrasound, China-Japan Friendship Hospital, Beijing, 100029, P.R. China.; 11 Shuwen Guo, Beijing University of Chinese Medicine, 11 Bei San Huan Dong Lu, ChaoYang District, Beijing 100029, P. R. China; 12 Ghulam Murtaza, Department of Pharmaceutical Sciences, COMSATS Institute of Information Technology, Abbottabad, Pakistan.; 13 Min Zheng, 1,4-10,13: Department of Ultrasound, China-Japan Friendship Hospital, Beijing, 100029, P.R. China.

**Keywords:** Ultrasonography, Color Doppler, Lower extremity artery, Diabetic vascular disease

## Abstract

***Objective:*** One of the major complications of diabetes is blood vessel disease, termed angiopathy, which is characterized by abnormal angiogenesis. The objective of this study was to discuss the characteristics of lower limb vascular angiopathy and plaque formation in type 2 diabetes patients and finding its relevance to the carotid atherosclerotic plaque formation, thus directing the clinical diagnosis and treatment.

***Methods:*** The ultrasonography was used to monitor the patients with carotid artery and lower limb artery.

***Results:*** Compared with the control group, decreased blood flow to lower limb and lower limb angiopathy occurred more obviously in dorsal artery of foot than in popliteal artery. *The study revealed that* the detection rate of the prevalence of carotid atherosclerosis plaque and lower limb arterial plaque and the combination of plaque both carotid and lower limb arteries in diabetic patients was 369:342:296 (about 1.25:1.15:1) and that the prevalence of carotid plaque and lower limb arterial plaque in all subjects with plaque was 71.3%. The risk of plaque formation also had positive correlation with patient’s age. Color Doppler ultrasound had a clinical significance in the early diagnosis and curative effect observation in type 2 diabetes with lower limb angiopathy. The risk of simultaneous plaque formation in both carotid artery and lower extremity artery was greater in type 2 diabetes than that of control subjects, but they were not necessarily to occur simultaneously. The symptoms were inconspicuous in the early course of diabetes.

***Conclusion:*** The application of ultrasound monitoring in patients with carotid artery and lower limb artery might play a role in early warning, delaying the occurrence of macrovascular diseases, and slowing down the development of macroangiopathy such as cerebral infarction and diabetic foot and so on, thus providing a significant basis for clinical diagnosis and treatment.

## INTRODUCTION

The incidence of lower limb angiopathy diseases in diabetic patients increases obviously which was the principal cause for the block or even amputation in lower limb artery.^1^^,^^2^ Therefore, it is the key point in preventing and managing diabetes to reduce diabetic chronic complications as far as possible. Recently, patients with carotid atherosclerotic plaque have been increasing, threatening the patient’s life and health. The study applies Color Doppler Ultrasound scanning, a kind of simple, convenient and feasible noninvasive technique, which can accurately locate and observe the characteristics of vascular lesions and the plaque formation in lower limbs of the patients with diabetes. Meanwhile, it can detect and differentiate the formation of carotid atherosclerotic plaque, find out the potential risk factors in patients with diabetes, and then provide basis for early clinical diagnosis and treatment as well as rehabilitation and intervention.

Thus the objective of this study was to discuss the characteristics of lower limb vascular angiopathy and plaque formation in type 2 diabetes patients and finding its relevance to the carotid atherosclerotic plaque formation, thus directing the clinical diagnosis and treatment.

## METHODS

***Subjects: ***The subjects were selected from the China-Japan Friendship Hospital of the Ministry of Health, including 2500 cases of out-patients from Ultrasonic Diagnosis Department and patients with type 2 diabetes and healthy people from body health center as well as 2350 cases of patients in observation of diabetes from November, 1999 to November, 2010.

As far as diagnostic criteria was concerned, the patients aged less than 88 years old and were in line with the type 2 diabetes mellitus (T2DM) diagnosis standard delivered by World Health Organization (WHO) in October, 1999. In addition, the patients without lower limb vascular diseases were excluded. Moreover, the patients with clinical cancer, infectious diseases and severe cerebrovascular diseases, and/or serious liver or kidney damages were also excluded.

A total of 583 eligible consenting patients entered the study including 262 male and 221 female patients aged from 25 to 85 years old and the average age (60 ± 13) years old. Their duration of disease lasted from 1 to 39 years and the average course was (9.6 ± 2.7) years. Likewise in the control group, there were 79 healthy male and 71 female patients aged from 27 to 81 years old, and the average age (57 ± 12) years old. Moreover, the age and gender of the two groups were comparable.

This study was approved by departmental ethical committee of China-Japan Friendship Hospital, Beijing, China and was carried out in accordance with the international guidelines for human use in clinical studies.

***Ultrasound examination methods:*** Spectral VS and Hitachi 6000 type produced by Disonic, USA was adopted. The angle between the ultrasonic beam and blood flow was less than 60° during the examination.

Two dimensional (2D) ultrasound was used for the detection of the condition of vascular wall, intima, cavity along the anatomical position through the visualization of the femoral artery, popliteal artery, anterior tibial artery, posterior tibial artery and dorsal pedal artery and then the endovascular blood flow and pipe cavity with Color Doppler Flow Imaging (CDFI) was observed to take samples from blood vessels with Pluse Width (PW) again to obtain the largest blood flow spectrum. The shape of the spectrum was also examined to determine the vascular internal diameter (D), peak flow velocity (Vmax), blood flow volume (Vol) and spectrum width (W), and calculate the artery stenosis degree in following formula:

Stenosis degree = (vascular internal diameter - vascular effective diameter) / vascular internal diameter×100%

The classifications of stenosis were as follows: type 0: there was no stenosis in blood vessels; type I: there was 1% to 19% of stenosis in the blood vessels; type II: there was 20% - 49%; type III: there was 50% to 99%. The last step was to view whether there was plaque in carotid artery and the lower limb arteries.

***S*** ***tatistical analysis:*** The study data was analyzed using SPSS17.0. Measurement data was expressed by mean ± standard deviation (X ± SD) and tested *t*, while enumeration data by frequency (%) and X^2^. *P* < 0.05 was considered statistically significant. 

## RESULTS

***C*** ***ondition of lower extremity vessels and blood flow in the control group:*** Lower limb artery wall consists of three layers i.e. intima, middle, and extima. The intima is of good smoothness and fine elasticity. The spectra of blood flow showed triphasic wave on pulse Doppler. The manifestations in diabetic limb angiopathy diseases were commonly thickened endarterium, lack or out of flatness and partial artherosclerotic plaques of different sizes protruding towards lumen which led to arterial-stenosis in different degrees or even block. The blood flow was significantly weakened in the stenosis with irregular morphous and the spectrum was broadened and filled, whereas normal triphasic wave was not found. Blocked vessel wall was thickened with inner cavity disappeared. Achromatic color blood flow showed that the spectra of blood flow failed, and the signal of blood flow in distal stenosis was weakened with lower flow of peak systolic velocity showed on Pulse Doppler (PD).

***Condition of lower extremity vessels and blood flow in the diabetic group:*** This study found that 699 blood vessels of diabetic patients (583 cases) damaged to some extent with 641 vascular lumen stenosis among which 84 were narrowed by 50%-99%, and 43 with vascular occlusion. Among 150 subjects in the control group, 43 blood vessels were found abnormal in 26 subjects taking up 17% in all subjects with 21 vascular lumen stenosis narrowed by less than 50% and 6 vascular lumen stenosis by 50%-77% in 5 subjects. As shown in Table-I, compared with the control group, the lower limb angiopathy occurred more obviously in dorsal artery of foot (P<0.01) than in popliteal artery(P<0.05); the blood flow was lower than that of the control group, especially in dorsal artery of foot (P<0.05);the blood flow velocity was accelerated more in dorsal artery of foot (P<0.01) than in popliteal artery (P<0.05); the blood flow spectrum had broadened in various degrees, more obviously in dorsal artery of foot than in popliteal artery (P<0.01). Therefore, the data from our study showed that the changes in the dorsal artery of foot were the most significant and all of the artery lesions were bilateral or plurisegmental for the lower extremity arterial diseases in diabetic patients (LEADDP).

**Comparison of** **carotid** **atherosclerosis plaque and lower limb plaque in patients with diabetes at different ages:** *The study revealed that* the detection rate of the prevalence of car*otid atherosclerosis plaque* and lower limb arterial plaque and the combination of plaque both carotid and lower limb arteries in diabetic patients was 369:342:296 (about 1.25:1.15:1) and prevalence of carotid plaque and lower limb arterial plaque in all subjects with plaque was 71.3%. Meanwhile, the prevalence of carotid atherosclerosis plaque and/or lower limb arterial plaque in type 2 diabetic patients increased with age.

## DISCUSSION

Carotid and lower limb vascular lesions were one of the most serious complications of diabetes showing no exact mechanism of disease pathology. Although there exists many factors of arterial sclerosis, the occurrence for the patients with diabetes increased obviously. One of the reasons might be as blood sugar raised, the oxidation irritability increased, and free radical spawns in large amounts, causing dysfunction of vascular tissue multicellular, which fortified the interaction between white blood cells and endothelium, the reaction of lower density lipoprotein oxidation and glycosylation and a series of chain reaction caused by the deposition of advanced glycosylation end products (AGE).^3^^,^^4^

**Table-I:** Comparison of the indexes of the blood rheology indices between the diabetic and control group (Mean ± SD).

*Position*	*D (mm)*	*V*_max _ *(m/s)*	*Vol (cm* ^3^ */s)*	*W*
*Left femoral artery (LFA)*				
Control group	7.41±0.69	0.85±0.25	41.2±6.79	5.08±0.67
Diabetes group	6.83±1.18	0.99±0.28	36.2±13.8	6.45±1.23
*Right femoral artery (RFA)*				
Control group	7.09±0.85	0.87±0.13	35.8±7.6	5.68±0.66
Diabetes group	7.12±0.95	0.90±0.15	36.5±16.8	6.66±1.24
*Left popliteal artery*				
Control group	5.90±0.68	0.51±0.09	13.6±3.3	4.29±0.73
Diabetes group	5.26±0.66^a^	0.66±0.23^a^	14.2±6.2	5.79±1.21^b^
*Right popliteal artery*				
Control group	5.70±0.59	0.53±0.16	13.7±3.4	4.71±0.72
Diabetes group	5.33±0.88	0.66±0.41	11.3±4.9	5.88±1.01^ a^
*Left dorsalis pedis artery*				
Control group	1.84±0.29	0.39±0.15	1.2±0.5	4.14±1.19
Diabetes group	1.38±0.38^ b^	0.45±0.22^ a^	0.7±0.5^ a^	5.77±1.28^ b^
*Right dorsalis pedis artery*				
Control group	1.99±0.44	0.39±0.15	1.5±0.7	4.08±1.02
Diabetes group	1.20±0.25^ b^	0.66±0.23^ a^	0.7±0.5^ a^	6.01±1.16^ b^

Compared with the control group,^ a^*P*＜0.05,^ a^*P*＜0.01

However, the state of continuous high blood glucose in diabetes accelerated the process of AGE and raised AGE forming capacity,^5^ contributing to the aggregation of AGE in patient's tissues, the proliferation of vascular smooth muscle cells, the incrassation or protrusion of vessel wall and reduction of vessel elasticity. The principal pathological changes in the neck and lower limb angiopathy diseases in patients with diabetes mellitus were the thickening of intima-media thickness (IMT), stenosing in vascular lumen, and reducing in the wall compliance.^6^^-^^8^ The formation of irregular atherosclerotic plaque in vascular intima resulted in further narrowing of lumen, then secondary thrombosis and even vascular occlusion.^9^^,^^10^ The vascular pathological changes of diabetes were similar to those of patients without diabetes. However, diabetes had a higher incidence and a faster progress of atherosclerosis, which might exist in the early period and thicken the intima-media membrane of carotid artery crotch and proximal internal carotid artery, and even cause plaque.^11^

The manifestations in diabetic lower extremity arterial diseases were commonly thickened endarterium, lack or out of flatness and partial artherosclerotic plaques of different sizes protruding towards lumen which led to arterialstenosis in different degree or even block. The blood flow was significantly weakened in the stenosis with irregular morphous and accelerated and the spectrum was broadened and filled, whereas normal triphasic wave was not found. Blocked vessel wall was reduced with inner cavity disappeared. Achromatic color blood flow showed that the spectra of blood flow failed, and the signal of blood flow in distal stenosis was weakened with lower flow of peak systolic velocity showed on Pulse Doppler (PD).

This study found that the blood vessel of diabetic patients damaged to some extent with 641 **vascular lumen stenosis cases among which 84 were narrowed by **50%-99%, and 43 with vascular occlusion. Among 150 subjects in the control group, 43 blood vessels were found abnormal in 26 subjects taking up 17% in all subjects with 21 **vascular lumen stenosis narrowed by less 50% and 6 vascular lumen stenosis by **50%-77% in 5 subjects. The blood vessel diameters were significantly reduced, and the blood flow volume was decreased and the blood flow rate was increased and the blood flow spectrums were broadened in various degrees in the diabetes group, compared with those in the control group, especially for that of the dorsal artery of foot, following by the popliteal artery. Therefore, the data from our study showed that the changes in the dorsal artery of foot were the most significant and the entire artery lesion was bilateral or plurisegmental for the lower extremity arterial disease in diabetic patient.

*In addition, the study also revealed that* the detection rate of the prevalence of carotid plaque and lower limb arterial plaque and both carotid and lower limb arterial plaque in diabetic patients was 369:342:296 (about 1.25:1.15:1) and that the prevalence of carotid plaque and lower limb arterial plaque in all subjects with plaque was 71.3%. Meanwhile, the prevalence of carotid plaque or／and lower limb arterial plaque in type 2 diabetic patients increased with age. The study suggested that the diabetic patients with the lower arterial diseases were more likely to suffer from the carotid plaque or／and lower extremity arterial plaque and should be examined earlier.

## CONCLUSION

In conclusion, the prevalence of carotid and lower limb angiopathy in diabetes mellitus is so high that severely threaten the quality of patient’s life. Although angiography is the gold standard for the diagnosis of vascular diseases at present, it is invasive and expensive. However, the color Doppler Ultrasound which is of high sensitivity, convenient inspection and good repeatability, is easier for patients to accept and ensures the early detection of the disease. It greatly increases the detection rate of lower limb angiopathy and is of important guiding significance for clinical early diagnosis, prevention and treatment of diabetic carotid and lower limb angiopathy diseases, making it a necessary inspection for diabetic patients with vascular tissue cell dysfunction. The application of ultrasound monitoring in patients with carotid artery and lower limb angiopathy plays a role in early warning, delaying the occurrence of macrovascular disease and slowing down the development of macro-angiopathy such as cerebral infarction and diabetic foot and so on, thus providing a significant basis for clinical diagnosis and treatment.
